# *EMinsight*: a tool to capture cryoEM microscope configuration and experimental outcomes for analysis and deposition

**DOI:** 10.1107/S2059798324001578

**Published:** 2024-03-26

**Authors:** Daniel Hatton, Jaehoon Cha, Stephen Riggs, Peter J. Harrison, Jeyan Thiyagalingam, Daniel K. Clare, Kyle L. Morris

**Affiliations:** a Data Analysis, Diamond Light Source, Harwell Science and Innovation Campus, Didcot OX11 0DE, United Kingdom; b Scientific Computing, Science and Technology Facilities Council, Harwell Science and Innovation Campus, Didcot OX11 0QX, United Kingdom; c Electron Bio-Imaging Centre (eBIC), Diamond Light Source, Harwell Science and Innovation Campus, Didcot OX11 0DE, United Kingdom; d Electron Microscopy Data Bank, European Molecular Biology Laboratory, European Bioinformatics Institute, Wellcome Genome Campus, Hinxton, Cambridge CB10 1SD, United Kingdom; STFC Rutherford Appleton Laboratory, United Kingdom

**Keywords:** *EMinsight*, cryoEM, data mining, deposition, machine learning

## Abstract

The widespread adoption of cryoEM technologies for structural biology has pushed the discipline to new frontiers. A significant worldwide effort has refined the single-particle analysis (SPA) workflow into a reasonably standardized procedure. Significant investments of development time have been made, particularly in sample preparation, microscope data-collection efficiency, pipeline analyses and data archiving. The widespread adoption of specific commercial microscopes, software for controlling them and best practices developed at facilities worldwide has also begun to establish a degree of standardization to data structures coming from the SPA workflow. There is opportunity to capitalize on this moment in the maturation of the field, to capture metadata from SPA experiments and correlate the metadata with experimental outcomes, which is presented here in a set of programs called *EMinsight*. This tool aims to prototype the framework and types of analyses that could lead to new insights into optimal microscope configurations as well as to define methods for metadata capture to assist with the archiving of cryoEM SPA data. It is also envisaged that this tool will be useful to microscope operators and facilities looking to rapidly generate reports on SPA data-collection and screening sessions.

## Abstract 2

*EMinsight* is a Python-based tool for systematically mining metadata from single-particle analysis cryoEM experiments. The capture and analysis of metadata facilitates the assessment of instrument performance, provides concise reporting of experiment performance and sample quality by analysing preprocessing results, and gathers metadata for deposition. It is envisaged that this approach will benefit the microscope operator, facility managers, database developers and users.

## 1. Introduction

Cryogenic-sample electron microscopy (cryoEM) has undergone significant growth and has matured into a major tool for determining the structures of macromolecular complexes at resolutions that are useful in structural biology research. This progress is evident in the substantial number of entries in the Electron Microscopy Data Bank (EMDB), which at the time of writing stands at 24 576 for single-particle analysis (SPA). The SPA technique involves using a transmission electron microscope (TEM) instrument to acquire many thousands of two-dimensional images of a target biological macromolecule preserved under cryogenic conditions and using computational techniques to identify the different poses of that macromolecule to reconstruct its three-dimensional structure. The growth of the technique can be attributed to improvements in various aspects of the SPA workflow, including sample preparation, automation and efficiency gains in data collection, and data-analysis techniques.

One crucial aspect that has gained prominence in enhancing microscope data-collection efficiency is the computerized control of microscopes. Several software packages, such as *Leginon* (Carragher *et al.*, 2000 ▸), *SerialEM* (Mastronarde, 2003 ▸) and *EPU* (*e pluribus unum*; out of many, one) from Thermo Fisher Scientific (TFS) have emerged as major tools in instrument control, allowing increased levels of data production via autonomy and lowering the technical barrier in controlling TEMs. At the time of writing, 72% (17 657) of the SPA macromolecular structures deposited in the EMDB are recorded as having been obtained using a Titan Krios microscope, as now produced by TFS, reflecting a standardization that has occurred due to the predominance of an instrument type in the field. The evident widespread adoption of instrumentation and collection strategies presents an opportunity to develop processes that attempt to standardize the capture of metadata from cryoEM imaging experiments. Such a process would benefit the community, enabling the automatic and robust generation of descriptions of how an experiment was performed. Such a tool to survey or parse instrument metadata also presents the opportunity for facilities to globally monitor the utilization and performance of their instruments.

**Figure 1:**
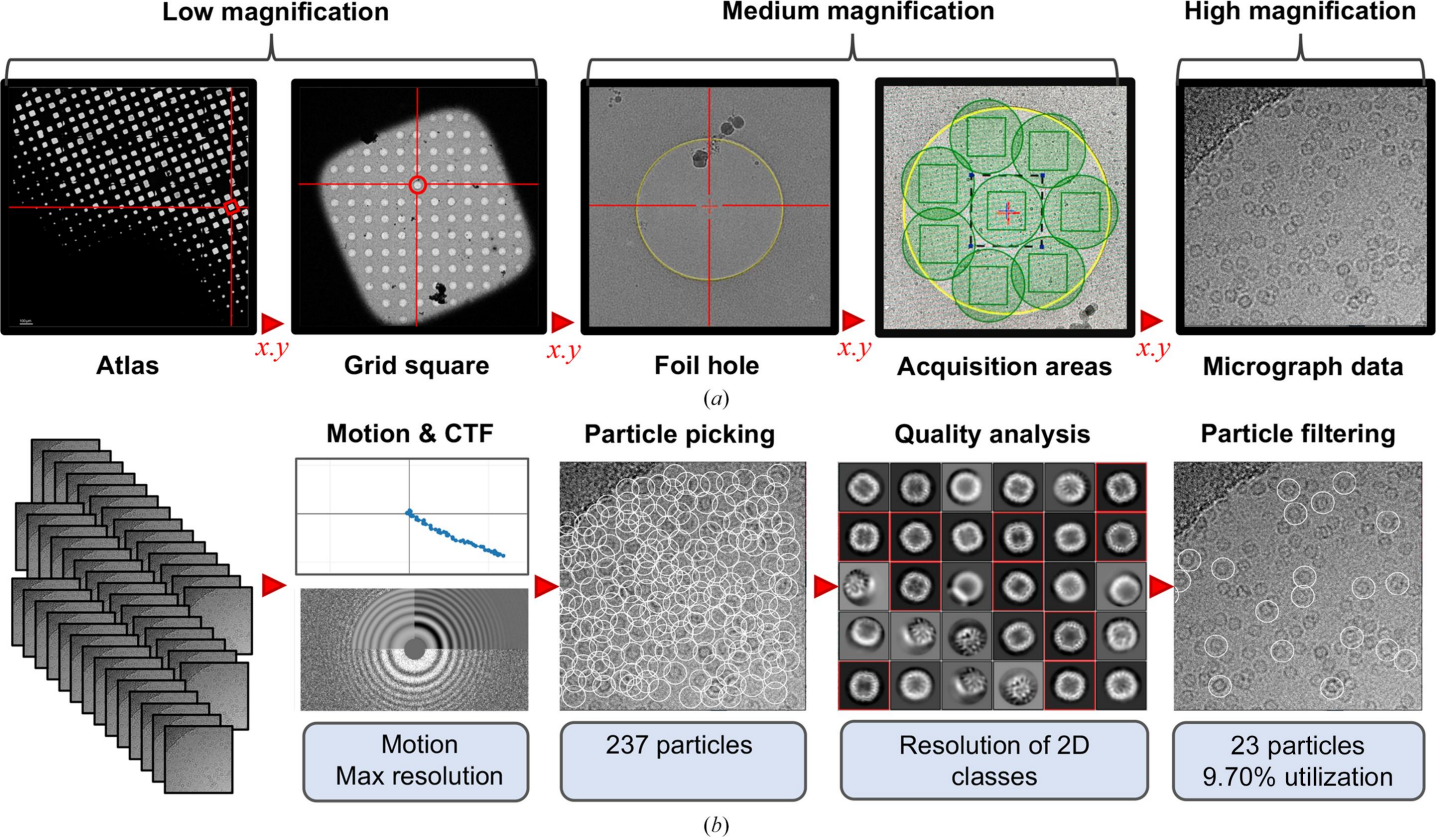
Workflow for an SPA cryoEM experiment showing (*a*) the hierarchical image-collection structure representing the collection strategy to arrive at the acquisition of micrograph data and (*b*) the preprocessing of many micrographs to arrive at filtered particles ready for structure determination. Some potential quality metrics that can be obtained from preprocessing are highlighted in light blue.

Fig. 1 ▸ graphically represents the SPA workflow at (i) the level of the experiment and (ii) in image processing. Acquisition workflows are reasonably well standardized in SPA. As the images (or micrographs) taken of the target macromolecules are destructive due to radiation damage, these data may only be collected from an area once. Thus, the experimental workflow may be thought of as a targeting exercise, whereby an expert operator uses nondestructive low-dose low-magnification images to identify regions of the specimen (atlas and grid square) expected to yield data of high quality. The knowledge of which regions produce high-quality data may have been established from prior knowledge gained during trial collections on the current or equivalent specimen (so-called ‘screening’), but in essence the goal of the operator is to set the microscope to target the coordinates of many foil holes and, within these, many acquisition areas. The microscope will then automatically collect micrograph data in those acquisition areas. This targeting exercise collects and produces a hierarchical image structure where acquired micrographs exist in relation to a series of lower magnification images that describe the location of that micrograph on the specimen.

Assessments of the quality of micrographs from SPA experiments are made as a product of the image processing that is performed to transform two-dimensional images into a three-dimensional structure of the target macromolecule. This is described in detail in various general reviews (Orlova & Saibil, 2011 ▸; Saibil, 2022 ▸; Lyumkis, 2019 ▸). Due to the broad range of software available for structure determination in SPA cryoEM, image-processing workflows can uniquely evolve for each structure-determination project. Increasingly, however, inline analysis packages are available to perform the image-processing steps leading to particle identification (so-called particle picking) and 2D alignment, averaging and classification (Punjani *et al.*, 2017 ▸; Fernandez-Leiro & Scheres, 2017 ▸; Gómez-Blanco *et al.*, 2018 ▸; Tegunov & Cramer, 2019 ▸; Caesar *et al.*, 2020 ▸), which we refer to as a preprocessing pipeline and depict in Fig. 1 ▸(*b*). Many packages are able to automatically perform analyses to produce three-dimensional reconstructions and the quality of the three-dimensional density is the *de facto* measure of the success of the experiment; however, preprocessing pipelines arguably already produce many of the metrics suitable for describing micrograph data quality. Benefitting again from a relative standardization of the preprocessing approach, there is an opportunity to capture these quality metrics as metadata describing the quality of an acquired data set of micrographs.

Taken together, these metadata describe how the experiment was configured and performed, and the experimental and analytical outcomes relating to the performance of the instrument and specimen. Many of these metadata points may still be manually documented by users, but could equally be retrieved from outputs from the instrumentation and pipelines that performed and analysed the experiment. If performed automatically, this would lead to efficiency gains for depositors and an increase in the robustness of the deposition process. The automatic capture of metadata describing the cryoEM experiment could also lower the barrier to capturing and depositing more descriptive data on the experiment. If available, this would permit hypothesis testing on the relationship between the experimental configuration and the experimental outcome. We would expect that an increased richness of metadata in the structural biology EM archives (Lawson *et al.*, 2016 ▸; Iudin *et al.*, 2023 ▸; Patwardhan & Lawson, 2016 ▸) will also make entries ready to support future machine-learning (ML) applications that require more descriptive labels for training and inference.

We present a tool, called *EMinsight*, which allows the systematic extraction of information from TFS *EPU* SPA directories to collate and summarize metadata describing the experiment. The directories of associated pipeline preprocessing in *RELION* are also interrogated to associate quality information with a collected data set. The outputs of *EM­insight* are expected to be useful for different end uses: PDF reports on the experiment for the microscope operator’s documentation, comprehensive metadata capture for facility database and instrument managers, and concise metadata capture with data-integrity measures for archive and deposition developers. We expect that this type of tool may form the conceptual basis for future systems that could be used locally within facilities to monitor instrument and session performance. Additionally, *EMinsight* could represent one type of approach to a method of generating metadata to automatically populate archival depositions of macromolecular structures, maps and raw data of SPA cryoEM experiments.

## 2. Materials and methods

### 2.1. Data structure

*EMinsight* has been developed with the SPA session type performed at eBIC (the UK national cryo-EM facility), Diamond Light Source. The software expects a data structure as shown in Table 1 ▸.

### 2.2. XML metadata parsing

Under the *EPU* SPA collection system (TFS), metadata describing the experiment are stored in XML format. These files are produced by *EPU* during an SPA session and store much of the metadata describing the configuration and behaviour of the microscope during the experiment. *EM­insight* has been developed at eBIC and *EPU* version 3.0. In general, every image that is acquired by the microscope and collection system is paired with an XML metadata file documenting the optical configuration of the microscope for that image as well as additional information. Information about the setup of the experiment is stored in hierarchical auxiliary files that do not necessarily have images associated with them (also in XML format file, with the extension .dm). For instance, the number and names of grids inventoried for collection is stored in a global ScreeningSession.dm but the individual grid type, hole diameter and radius are stored in an EpuSession.dm for each grid. *EMinsight* uses a Python library to transform the XML into a dictionary and then query known addresses for instrument and experimental metadata. In general, these are passed to the *pandas* Python library to create internal dataframes for reference, analysis and output.

### 2.3. Preprocessing analysis parsing

During SPA collections at eBIC, an automatic preprocessing pipeline is executed using *RELION* and components of the *CCP-EM* pipeliner (https://gitlab.com/ccpem/ccpem-pipeliner). This includes motion correction, CTF estimation, particle picking using *crYOLO* (Wagner *et al.*, 2019 ▸), 2D classification, particle selection and subsetting. The directory structure of the results is as is typically expected for *RELION*, including a relion_it_options.py file containing many of the parameters defining the processing pipeline. *EMinsight* reads files in the expected *RELION* directory structure (tested using *RELION* version 4.0 and *CCP-EM* pipeliner version 0.1.0) and parses some elements of the relion_it_options.py file to conveniently gather the pipeline parameters and results and associate them with each individual micrograph of a data set. Associations of those micrographs with their respective originating grid square and hole locations are retained such that they can be used for data grouping in location-based analyses.

### 2.4. Particle-picking analyses

Particle picking at eBIC is typically performed using *crYOLO* (Wagner *et al.*, 2019 ▸). This is leveraged to read the particle diameter by parsing and averaging the particle diameters reported for each picked particle by *crYOLO* in the output *.cbox files. While keeping in mind a data set with large amounts of carbon or ice contamination may interfere with the interpretation of particle-picking analysis, *EMinsight* measures particle density and particle clustering. These are calculated independently, allowing the user to identify particle-distribution pathologies. Particle density is calculated as a value normalized to 1 and considers only picks made with a *crYOLO* score over a threshold of 0.3 in an attempt to exclude false-positive picks (Wagner *et al.*, 2019 ▸). A density of 1 would represent maximum packing of particles into a simplified 2D array in the micrograph field of view based on the observed particle diameter. In brief, the circular diameter of a particle is used to approximate the area of the particle to a square with that diameter. The ideal packing density is then estimated as the number of particles given by dividing the total magnified sensor area by the approximated particle area. Particle coordinate clustering is calculated independently of particle density using the *scikit-learn* implementation of nearest-neighbour analysis. Where the nearest-neighbour distance is found to be less than 80% of the measured particle diameter, the particle is labelled as overlapping or, as termed in *EMinsight*, clustered.

### 2.5. Outputs

Each of the following subsections describes the outputs that a user of *EMinsight* can expect to be produced.

#### 2.5.1. Comma-separated value (CSV) collated data

*_datastructure.csv: associates the micrographs with the associated lower magnification images, as well as with quality metrics gathered from metadata.

*_optics.csv: reports all of the microscope optics configurations for each preset used by *EPU* for the data-collection session.

*_processed.csv: reports on the outcomes of preprocessing jobs to infer the quality of the data set.

*_session.csv: reports on the outcomes of the session, *i.e.* targeting statistics, collection rates, data-set size, specimen properties and collection strategy.

#### 2.5.2. Reports

PDF reports are created to summarize and present the major descriptors of the data-collection session to the end user of *EMinsight*. These are *_session.pdf to present information on the session configuration and outcome and *_processed.pdf to present information on the preprocessing outcomes.

#### 2.5.3. Deposition

JSON files recording the necessary fields for populating the mandatory fields of the Microscopy section of an EMDB deposition. Checksums are included to provide a method of verifying data integrity.

## 3. Results

### 3.1. Reporting on SPA sessions

**Figure 2:**
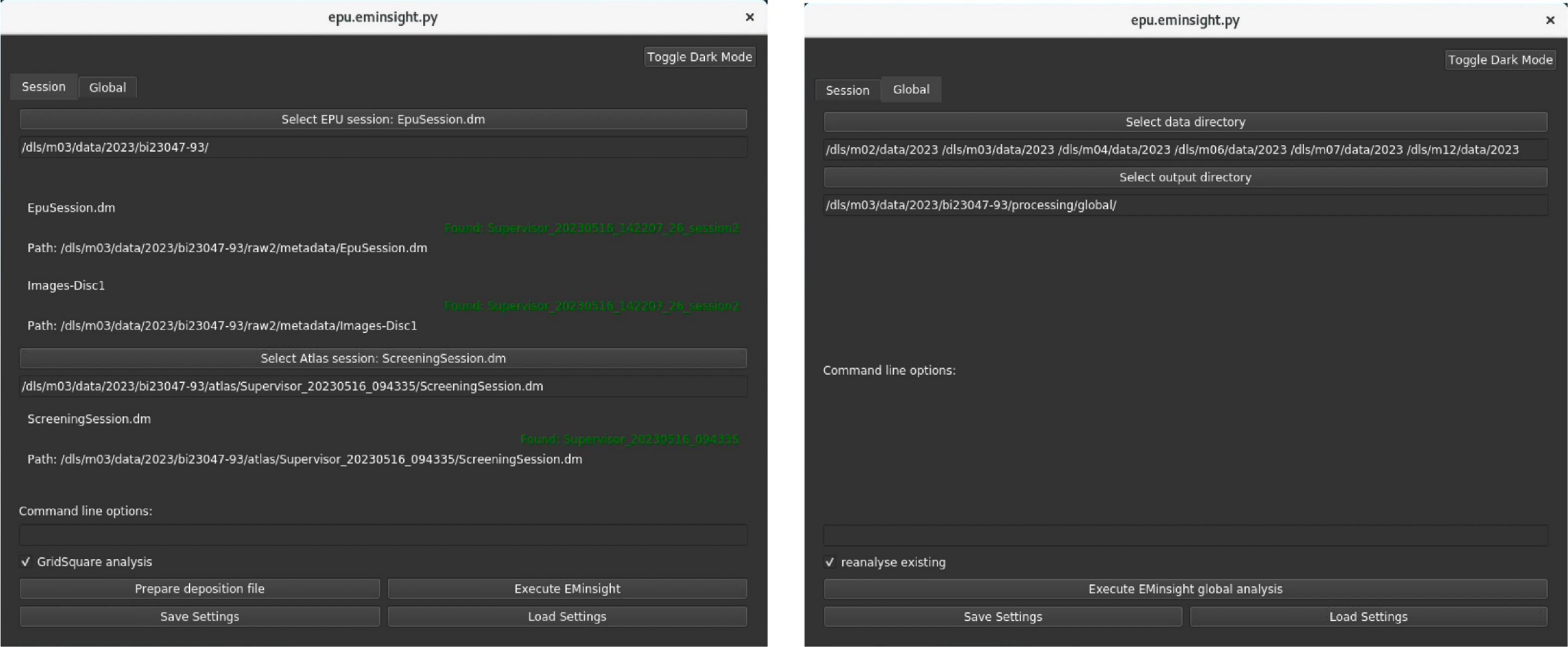
The *EMinsight* user interface (left) for analysing a single cryoEM SPA data-collection session and (right) for analysing and collating session analyses across multiple directories from an SPA user programme.

All raw data and the hierarchical image structure from SPA experiments are written out by *EPU* along with metadata in XML format. These can be interrogated to expose the configuration of the experiment; however, these metadata are not practically human-readable. *EMinsight* parses through the metadata structure of experimental outputs from *EPU* sessions to gather important data describing the experiment to produce a concise human-readable report. *EMinsight* can be executed from the command line or a simple user interface, as shown in Fig. 2 ▸.

Example PDF reports are shown in Supplementary Section S1 and are expected to be useful as reference documents when stored as part of an electronic laboratory notebook by the microscope operator and *EMinsight* user. The types of metadata that are captured and exposed by *EMinsight* are summarized in Table 2 ▸. In addition to capturing Instrument Configuration metadata, additional properties of an SPA session may be described as Experimental Outcomes, Calculated Parameters and Analytical Outcomes. Many of these descriptors are displayed in the PDF reports, but all exposed descriptors are written to CSV files serving as a simple collated data for each session, as shown in Supplementary Section S2.1. *EMinsight* is further capable of parsing multiple experiments and globally aggregating collated data, as shown in Supplementary Section S2.2.

### 3.2. Instrument-performance assessments

**Figure 3:**
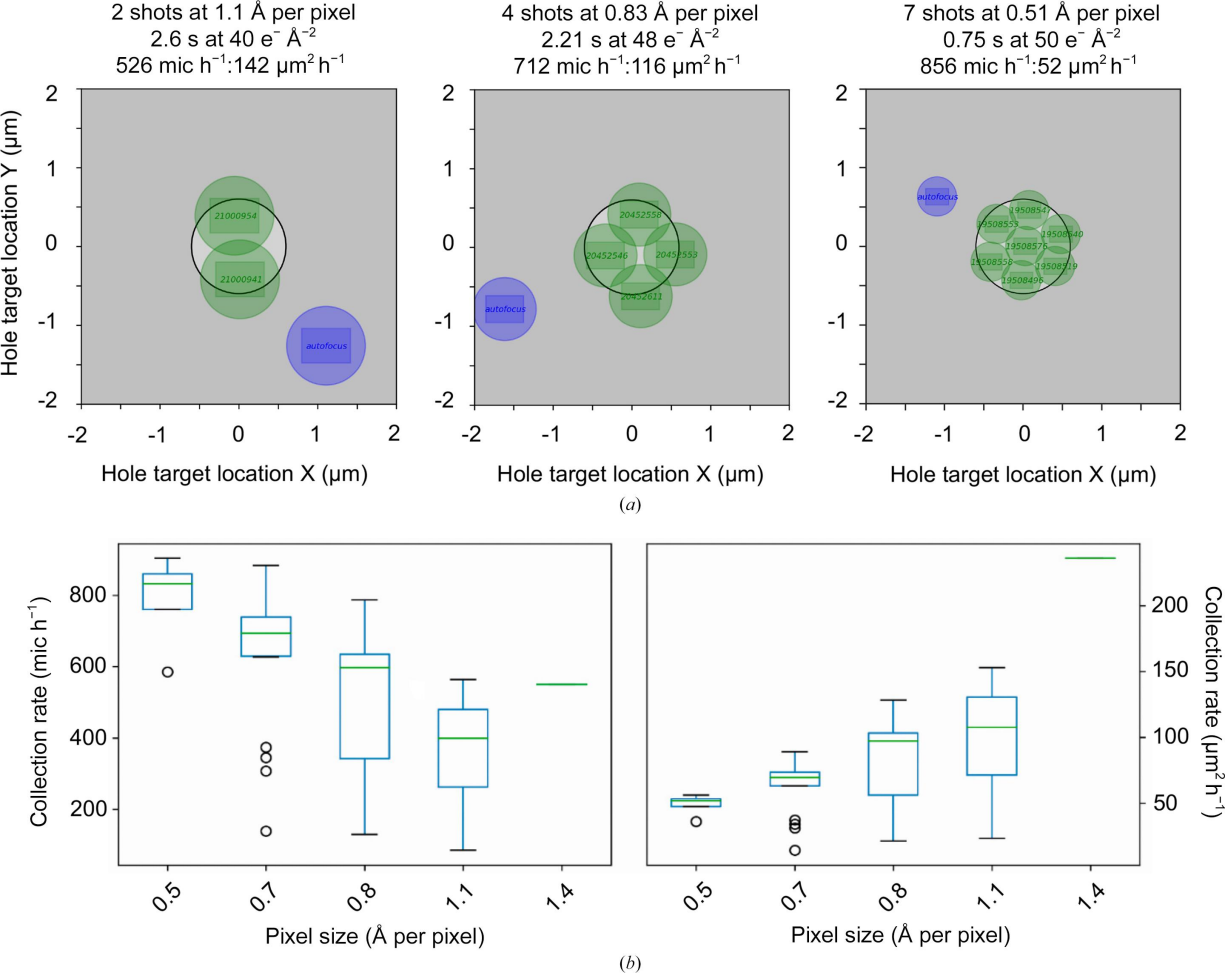
Multi-shot data-acquisition approaches achievable by increasing the microscope magnification. Representations are shown in (*a*) with their session-associated collection performance on a Titan Krios equipped with a Gatan K3/BioQuantum camera/filter system. (*b*) The collection performance in micrographs per hour (mic h^−1^) and µm^2^ h^−1^ for the same Titan Krios microscopes with a Gatan K3/BioQuantum camera/filter system is expressed as boxplots, robustly revealing that the speed gains that are obtained from increasing magnification do not offset the loss in field of view.

*EMinsight* performs systematic analyses on individual data-collection sessions as well as comparing these analyses across multiple sessions from a cryoEM instrument as part of a facility or user programme. In one example, this type of analysis confirms the speed gains that can be attained on an eBIC microscope (TFS Titan Krios) by increasing the magnification and employing multi-shot collection facilitated by aberration-free image shifting (AFIS). However, it is important to note that these speed gains are not sufficient to offset the loss of field of view incurred due to the increase in magnification (Fig. 3 ▸ *a*). A systematic analysis of collection rates across multiple Titan Krios instruments confirms this behaviour (Fig. 3 ▸ *b*), where multiple strategies may have been employed to enhance collection rates but are still unable to collect the same amount of usable area as the magnification is increased. In the light of this, microscope operators might first consider the resolution that they need to achieve, using the largest pixel size appropriate for this and then carefully assessing how long they need to collect given expected data-collection rates with a particular magnification and experimental setup. Considering efficient data collection using larger pixel sizes has been suggested elsewhere (Harrison *et al.*, 2023 ▸). *EMinsight* provides a means to empirically assess the data-collection rates that can be expected on an instrument in a particular configuration, allowing these calculations to be driven by historical performance data. Indeed, a Titan Krios that has a significant configuration difference in its detection system performs differently (see Supplementary Fig. S1) to the analyses presented in Fig. 3 ▸. As vendors improve instrument and data-collection workflows, further gains in speed may be realized. In the meantime, the community may want to consider utilizing the gains in speed and area achievable from low-magnification collection (*i.e.* 1.5 Å per pixel; super resolution 0.75 Å per pixel) in combination with super-resolution camera detection modes to allow the recapitulation of high-resolution information.

### 3.3. Experimental performance assessments

**Figure 4:**
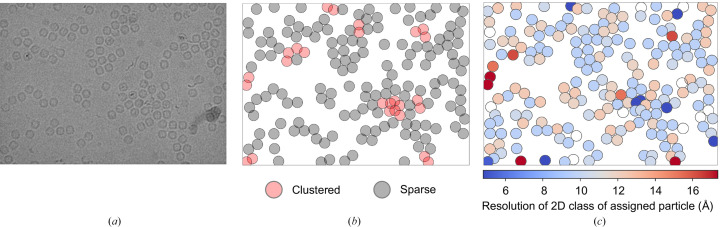
A representative set of particle-coordinate analyses showing (*a*) micrographs, (*b*) the associated picks, where clustered particles are represented in red, and (*c*) the resolution of 2D classes to which particles within a micrograph are assigned.

As automatic processing pipelines increasingly become adopted at cryoEM facilities, it is possible to rapidly inform the operator of the quality of their sample (Punjani *et al.*, 2017 ▸; Fernandez-Leiro & Scheres, 2017 ▸; Gómez-Blanco *et al.*, 2018 ▸; Caesar *et al.*, 2020 ▸) and critically to provide feedback with increasing detail and as early as possible during experimental time. Pipeline implementations may be customized by the facility to suit the local compute infrastructure, but off-the-shelf solutions are publicly available in packages such as *RELION* (Fernandez-Leiro & Scheres, 2017 ▸), *cryoSPARC* (*Live*) (Punjani *et al.*, 2017 ▸), *Scipion* (Gómez-Blanco *et al.*, 2018 ▸) and *Warp* (Tegunov & Cramer, 2019 ▸). Many of these pipelines attempt to perform analyses all the way to a three-dimensional reconstruction without user intervention; however, preprocessing pipelines are more commonplace in facilities. In this manuscript, reference is made to a pipeline that performs preprocessing steps including motion correction, CTF estimation, particle picking and initial 2D classification and averaging. Fig. 4 ▸ shows typical picked-particle coordinates for a micrograph as shown to the user in the *EMinsight* report and frequently reported by common image-processing software. *EMinsight* additionally performs a density and clustering analysis, as well as labelling each particle with the resolution of the 2D class that it is assigned to during preprocessing. We note that the *crYOLO* particle picker used already excludes some particle picks found in clusters and thus cases exhibiting severe particle clustering may be underestimated. These particle-quality metrics can then be averaged to describe each micrograph quality as a singular value. Whilst this averaging may hide subtle trends in the data, it is expected that these metrics will be useful for reporting global trends across data sets analysed by *EM­insight*.

**Figure 5:**
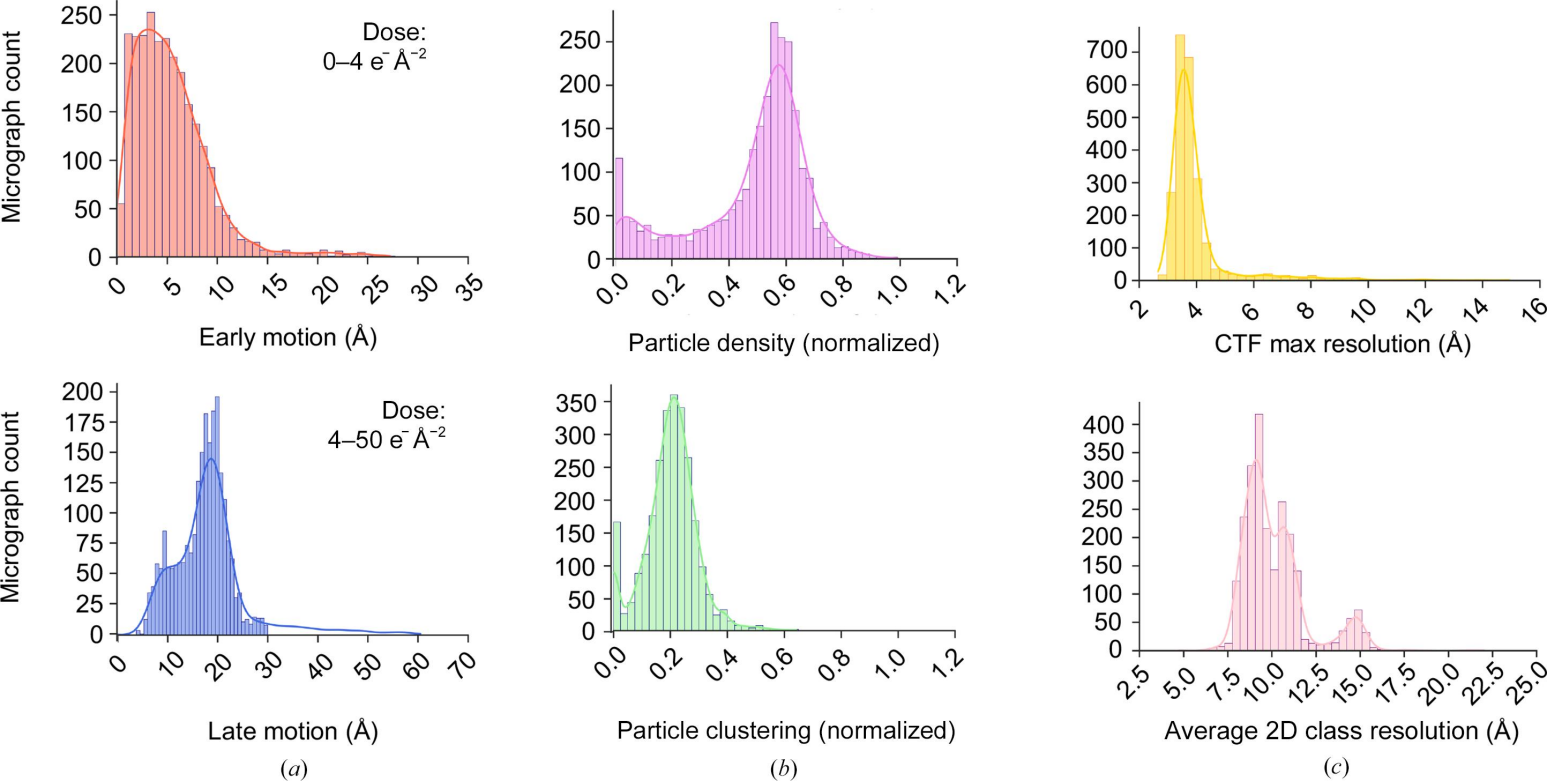
A representative set of analyses of preprocessing results prepared by *EMinsight* showing (*a*) specimen motion early (top) and late (bottom) during an image acquisition with a total dose of 50 e^−^ Å^−2^, (*b*) normalized particle density for a particle with a measured diameter of 136 Å (top) and degree of particle clustering where particles are closer than 109 Å (bottom), and (*c*) micrograph CTF maximum resolution (top) and the mean resolution of the 2D classes to which particles from a micrograph are assigned.

The particle-quality metrics are stored along with specimen motion (early/late/total) and CTF maximum resolution for each micrograph of the data set in an attempt to concisely represent the quality and variation in the whole SPA experiment. Specimen motion is characterized using the default *RELION* convention, where the cumulative motion in ångströms up to a dose of 4 e^−^ Å^−2^ is characterized as early motion and the cumulated motion after this dose to the end of the exposure as late motion. Fig. 5 ▸ shows the representation of these quality metrics for a single SPA experiment. The preprocessing results captured by *EMinsight* will reflect the quality of the specimen, but may also be influenced by the performance of the instrument in recording high-quality information on the specimen and so should be considered on a case-by-case basis. Where the collated data CSV outputs of *EMinsight* connect preprocessing results with instrument, experimental and derived metadata describing an SPA experiment, it may be possible to investigate whether particular instrument configurations are deterministic in pre­processing pipeline outcomes. *EMinsight* thus provides a way for microscope operators, facilities or data scientists to rapidly quantify and identify sessions that were experimentally successful and provides a framework for investigating how the instrument and specimen may together influence experimental success.

### 3.4. Analytical outcomes linked to specimen location

**Figure 6:**
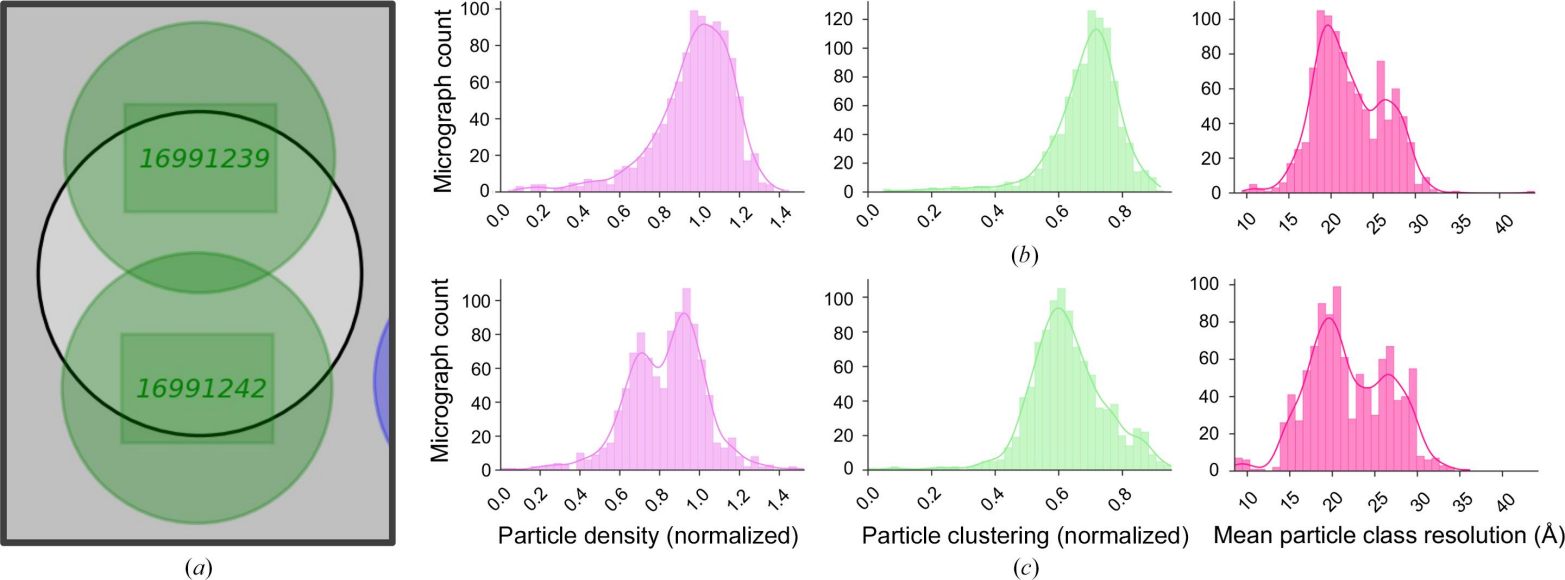
Analysing experiment performance in the context of instrument location metadata reveals trends in the particle density, aggregation and quality of particles in an SPA specimen. (*a*) The two exposure areas targeted for collection. The statistics for all exposures taken in the hole location for the top exposure (*b*) are shown in comparison to the bottom exposure (*c*).

Every micrograph name is stored by *EMinsight* in a way that allows the identification of all low-magnification images used to locate that target on the specimen. Additionally, the characteristics of each micrograph from the preprocessing pipelines are exposed by *EMinsight* and stored in the context of their locating atlas, grid square, hole and hole acquisition area image. Thus, micrograph characteristics from preprocessing pipelines can be displayed for specific areas of the grid. Metadata and preprocessing results may be analysed at various hierarchical levels of the imaging experiment. For instance, aggregating all data would reveal the overall behaviour of the specimen with respect to the entire grid or atlas. Data can then be separated at the level of the grid square or the data-acquisition area within a hole of the specimen support. At the level of the grid square the user may learn about the variability in the sample due to large variations in vitreous ice properties from the plunge-freezing process, whereas at the level of shots per hole a user may learn about the behaviour of the specimen inside the hole of the specimen support. Analysing the behaviour of the specimen inside the hole may be particularly interesting if a user could learn from this analysis to retarget their data collection. Fig. 6 ▸ shows an analysis of particle behaviour and quality from all micrographs of a data set separated into those exposures taken at the top or bottom of the foil hole. The resolutions of the 2D classes to which individual particles are assigned ultimately suggests that the particles are of equivalent quality in both target areas, despite differences in packing density and clustering. However, for data sets that exhibit pathological problems in particle distribution in holes it is expected that this type of analysis could be beneficial to retarget the data collection to collect higher quality data.

### 3.5. Instrument setup, performance and experimental outcome described in one database

The data structure at eBIC separates experimental visits by instrument, year, user group and visit number, as shown in Table 3 ▸. Due to the predictable data structure at eBIC and given the established methodology for parsing a single *EPU* SPA experimental metadatum, it is possible to systematically query every experimental visit on an instrument, for a particular user group or across the whole user programme. All of these data can then be aggregated to analyse trends across multiple data collections.

**Figure 7:**
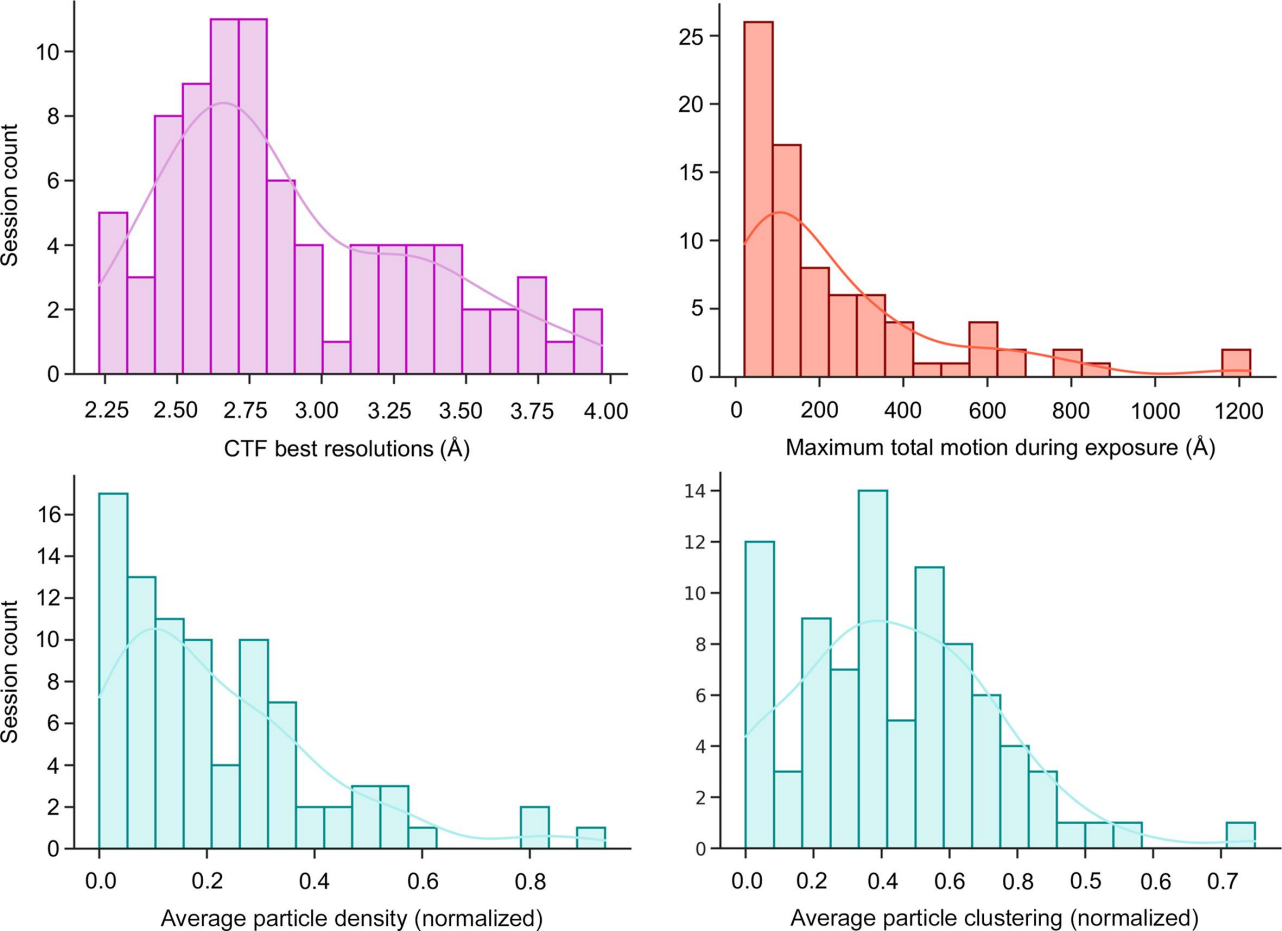
Several common performance metrics for cryoEM SPA experiments assessed across several months of a cryoEM Krios user programme.

Fig. 7 ▸ depicts several common performance metrics recorded from SPA cryoEM experiments. *EMinsight* performs data reduction to produce a single value, either as an average or a maximum/minimum value to describe a collection session. Whilst these metrics will vary for each micrograph within each data collection itself, histograms of the single reduced values aim to provide a rapid overview of the performance of an instrument or user programme. The CTF best resolutions are most commonly less than 3 Å; many sessions are subject to high levels of specimen motion, but the trend is towards data exhibiting motion of less than 200 Å. At the level of the specimen, most data sets are collected with micrographs exhibiting particles at less than ideal occupancy (optimal would equal a normalized value of 1) and most commonly data sets on average have low levels (∼20%) of particles found to be clustered, but all data sets suffer from a degree of particle clustering.

### 3.6. Deposition-ready data

*EMinsight* reinforces the concept for configuration files that can inform image processing or even deposition, as has been developed by other software packages (Gómez-Blanco *et al.*, 2018 ▸; Kimanius *et al.*, 2021 ▸). From metadata that are captured and exposed by *EMinsight*, as reported in Table 2 ▸, a subset is extracted and stored with a view to be useful for the deposition of SPA data and three-dimensional reconstructions in the archives. *EMinsight* prepares a single JSON file with many of the data fields that are necessary to populate the ‘Experiment: Microscopy’ sections of an EMDB archive entry. A checksum file is produced as a method to verify the integrity of the data within the deposition JSON file. Table 4 ▸ shows the deposition file fields generated by *EMinsight*.

### 3.7. Machine learning-ready data

As described previously, each micrograph that is assigned quality metrics is stored in a way that associates that micrograph with the lower magnification images in the hierarchical image structure taken to target that micrograph (see Section 2.5.1). *EMinsight* then introduces the concept that metadata describing high-magnification images could be used as quality labels for lower magnification images that would have been collected prior to high-magnification acquisition. This could be leveraged to allow the application of ML techniques in recognizing features in low-magnification images that lead to high-quality data acquisition in micrographs.

Further, the automatic collection of metadata sufficient for the experimental section of an EMDB deposition by *EM­insight* may represent one potential avenue towards inter­facing with EMDB (Lawson *et al.*, 2016 ▸) and EMPIAR (Iudin *et al.*, 2023 ▸), perhaps in future deposition procedures. Table 4 ▸ reports the fields that are collected by *EMinsight*. Some of these fields are not currently stored in archive depositions, but these extended metadata fields may prove to be relevant for increasing the descriptive power of an EMDB deposition entry. All in all, *EMinsight* could represent the type of end-user tool that is required to minimize the barrier to data submission to archives, whilst also maximizing opportunities for data reuse through metadata deposition carefully considered within recommended frameworks (Sarkans *et al.*, 2021 ▸). We envisage this to greatly benefit future ML projects relying on open-access, accurate and descriptive metadata of database entries.

## 4. Discussion

The widespread use of software-based solutions for inter­action with the electron microscope has improved the efficiency of data collection and enabled almost continuous automated instrument utilization. Despite these advances, there is still a need for effective record-keeping of instrument and experimental setups, especially given the complexity of SPA experiments. An SPA cryoEM experiment is often fully described in the metadata output by the instrument itself; however, these are not practically human-readable. *EMinsight* addresses this by converting intricate metadata into human-readable reports, aiding in the documentation of SPA experiments and potentially assisting with deposition to archives.

The capture of instrument configuration and experimental outcomes to derive calculated parameters then presents the opportunity for systematic analyses into factors affecting instrument performance. *EMinsight* offers insights into instrument performance by drawing from recorded metadata, but can further make inferences about the quality of the experiment and specimen by analysing preprocessing metadata. By capturing location data, analysis at various levels is enabled, such as a grid square or exposures within a hole, to identify issues with cryoEM SPA specimens. These tools help to correlate instrument configurations with experimental outcomes, which is expected to be useful to microscope operators, *EMinsight* users and facility managers in evaluating session success.

For deposition, *EMinsight* facilitates the recall of experimental details, which could be leveraged for populating metadata fields in structural biology archives such as EMDB and EMPIAR. When *EPU* has been used for data collection, *EMinsight* can produce files that could support the parsing of metadata in preparation for deposition, in a concept analogous to harvesting data from structure-determination applications in X-ray crystallography (Yang *et al.*, 2004 ▸; Potterton *et al.*, 2018 ▸). The development of automatic deposition workflows for cryoEM, possibly involving archive-deposition APIs, is anticipated to benefit from the deposition files produced by software such as *EMinsight*, but the functionality could also be incorporated into other software such as *Scipion* (Gómez-Blanco *et al.*, 2018 ▸) or *CCP-EM* (Burnley *et al.*, 2017 ▸). However, the heterogeneity in data-collection software and processing pipelines, and the potential for existing local procedures in metadata capture and storage in laboratory information-management systems already having been applied, increases the complexity in creating a unified system for metadata capture and automatic deposition. *EMinsight* is then representative of what is possible, but must be considered as an example of what could be performed rather than a final solution to this problem, which ultimately will require coordination from major instrument manufacturers, software developers and database developers.

Altogether, *EMinsight* represents a tool that gathers and relates instrument configuration, experimental outcomes and analytical outcomes in concise reports for immediate and historical analysis of SPA data-collection sessions. It could equally be adapted as a standalone tool or be incorporated into systems that feed back on cryoEM SPA experiments in real time. The coordinated recording of metadata of various kinds allows global analyses of the performance of instruments and the user programmes that they run. As a tool that interprets and exposes the hierarchical image structure of a cryoEM SPA experiment, each micrograph is related to its low-magnification images along with quality metrics and could be used as a precursor for training neural networks to recognize high-quality collection areas from low and medium-magnification images.

We envisage that software such as *EMinsight* will incentivize the retention of metadata produced by cryoEM instrumentation performing SPA experiments or derived databases that describe how experiments were performed. This will improve the ability of scientists to more easily recall how experiments were performed and what their outcomes were as they develop structural biology projects aiming to determine the structures of macromolecules. These types of software could robustly inform downstream analytical processes of microscope configurations and metadata describing the experiment to automatically run image analyses. In particular, we envisage that microscope metadata could be extracted automatically at the point of map deposition or associated and retained throughout image processing to then be ready to automatically populate the archives at the time of map deposition. More descriptive metadata in the structural biology archives themselves could facilitate better understanding of the relationship between how an SPA experiment was performed and the quality of the resulting map, as well as enabling future ML applications on archived cryoEM data.

## 5. Data availability

A representative data structure on which *EMinsight* can be run to reproduce the analysis in this manuscript has been uploaded to EMPIAR under the accession code 11895.

## 6. Source-code availability

*EMinsight* is available in the repository at https://github.com/kylelmorris/EMinsight and is distributed under the BSD-3-Clause licence.

## Supplementary Material

Supplementary Figure, report examples and links to supplementary files. DOI: 10.1107/S2059798324001578/qi5006sup1.pdf

## Figures and Tables

**Table 1:** A representation of the directory data structure expected by *EMinsight*

Directory structure	Notes
**Unique-session-identifier**	
**|- atlas**	
**| |- Supervisor_[date]_[time]-session-identifier_Atlas**	*EPU* atlas directory
| | |- ScreeningSession.dm	Screening metadata
**| | |- Atlas**	
| | | |- Atlas.dm	
**| | |- Sample1**	
| | | |- Sample.dm	
**| | | |- Atlas**	
| | | | |- Atlas.dm	Atlas metadata
| | | | |- Atlas.jpg/mrc	Atlas montage
| | | | |- Atlas.xml	Atlas metadata
**|– processed**	
**| |– raw**	
**| | |- relion**	Preprocessing results
**|– processing**	
**| |– gain**	
| | |- README	Facility metadata
**|- raw**	
**| |- GridSquare_[Square-ID]**	
**| | |- Data**	Raw data for grid square
| | ||-FoilHole_[Hole-ID]_Data_[Acquisition-ID]_[date]_[time]_fractions.mrc	Raw data image
**| |- metadata**	
**| | |- Supervisor_YYYYMMDD_HHMMSS_unique-session-identifier_EPU**	
**| | | |- Images-Disc1**	
**| | | | |- GridSquare_[Square-ID]**	
| | | | | |- GridSquare_[date]_[time].jpg/mrc	Grid-square image JPG/MRC
| | | | | |- GridSquare_[date]_[time].xml	Grid-square image metadata
**| | | | | |- Data**	
| | | | | | |- FoilHole_[Hole-ID]_Data_[Acquisition-ID]_[date]_[time].jpg/mrc	Data-image sum JPG/MRC
| | | | | | |- FoilHole_[Hole-ID]_Data_[Acquisition-ID]_[date]_[time].xml	Data-image metadata
**| | | | | |- FoilHoles**	
| | | | | | |- FoilHoles_[Hole-ID]_[date]_[time].jpg/mrc	Foil-hole image JPG/MRC
| | | | | | |- FoilHoles_[Hole-ID]_[date]_[time].xml	Foil-hole image metadata

**Table 2:** A selection of the metadata captured and exposed by *EMinsight* to the user

Instrument Configuration	Experimental Outcomes	Calculated Parameters	Analytical Outcomes
C2 aperture size	Total available squares	Dose rate (e^−^ Å^−2^ s^−1^)	Motion (early/late/total)
Spot size	Targeted squares	Rate (micrographs per hour)	CTF resolution (min/max)
Beam size (µm)	Collected squares	Rate (µm^2^ h^−1^)	Particle size
Magnification (×)	Collected images		Particle count
Defocus range (µm)	Collected area (µm)		Particle density
Grid type	Acquisition time stamps		Particle clustering
Hole size/spacing (µm)	Acquisition locations		Class assignment resolution
Shots per hole	Acquisition data structure		

**Table 3:** Global data structure for experimental visits at a large user facility

Directory structure	Note
**Instrument-unique-identifier**	
**|- year**	
**| |- Data**	
| | |- [userID-visitNumber]	Directory data structure depicted in Table 1 ▸

**Table 4:** An example of the fields populated by *EMinsight* in an example deposition file with extended metadata expected to be beneficial to complement those currently required for an EMDB archive deposition

Microscope	TITAN52334150
epuversion	3.0.0446.0
date	YYYY/MM/DD
eV	300000
Mag	81000
Apix	2.3
nominal_defocus_min_microns	−1
nominal_defocus_max_microns	−3
spot_size	5
C2_micron	50
Objective_micron	100
beam_diameter_micron	1.1
Collection	AFIS
number_of_images	254
grid_type	HoleyCarbon
available_squares	62
collected_squares	3
average_foils_per_square	140
hole_size_micron	1.2
hole_space_micron	1.3
shots_per_hole	2
total_dose_eA2	34.6
fraction_dose_eA2	0.6
