# Supplementary material for: EMinsight: a tool to capture cryoEM microscope configuration and experimental outcomes for analysis and deposition
Source: Acta Crystallogr D Struct Biol. 2024 Mar 26;80(Pt 4):259–69. doi: 10.1107/S2059798324001578 (PMC10994178; doi:10.1107/S2059798324001578)
Supplement: Supplementary file 1 [file d-80-00259-sup1.pdf]

STRUCTURAL BIOLOGY

Volume 80 (2024)

Supporting information for article:

EMinsight: a tool to capture cryoEM microscope configuration and experimental outcomes for analysis and deposition

Daniel Hatton, Jaehoon Cha, Stephen Riggs, Peter J. Harrison, Jeyan Thiyagalingam, Daniel K. Clare and Kyle L. Morris

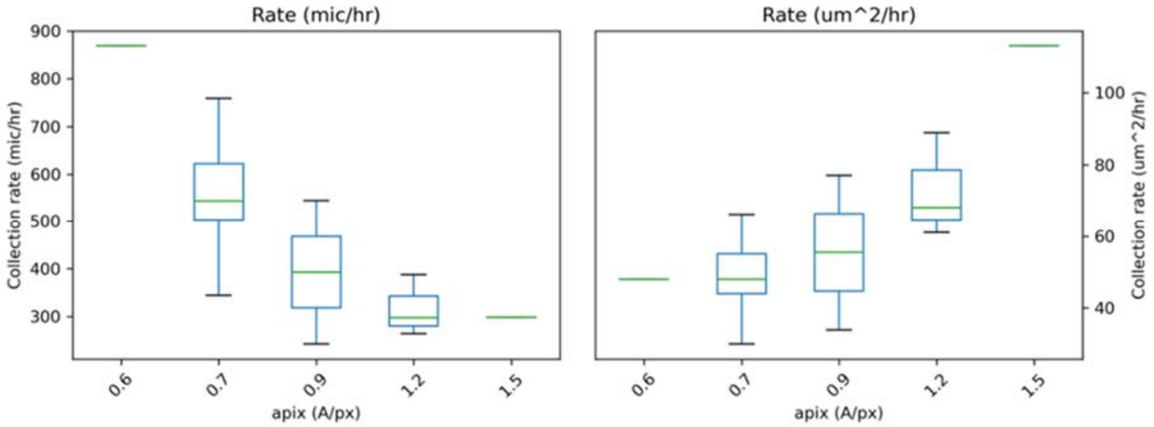

Figure S1 The collection performance in mic/hr and um^2/hr for three Titan Krios microscopes with Falcon 4i and Selectris X camera/filter systems.

S1. EMinsight session report example

The following report is produced by EMinsight describing a single SPA cryoEM experiment at the instrument performance level.

https://github.com/kylelmorris/EMinsight/blob/publication/expected_outputs/single_session/EMinsight/report/Supervisor_20230919_140141_84_bi23047-106_grid1/Supervisor_20230919_140141_84_bi23047-106_grid1_session.pdf

S1.1. EMinsight processed report example

The following report is produced by EMinsight describing a single SPA cryoEM experiment at the analytical performance level.

https://github.com/kylelmorris/EMinsight/blob/publication/expected_outputs/single_session/EMinsight/report/Supervisor_20230919_140141_84_bi23047-106_grid1/Supervisor_20230919_140141_84_bi23047-106_grid1_processed.pdf

S2. Supplemental files

S2.1. EMinsight single session expected outputs

The following are representative of the files that are produced by EMinsight to store metadata on a single SPA cryoEM experiment and are referenced to produce the PDF reports

S2.1.1. Collated data files

https://github.com/kylelmorris/EMinsight/tree/publication/expected_outputs/single_session/EMinsight/csv

S2.1.2. Deposition files

https://github.com/kylelmorris/EMinsight/tree/publication/expected_outputs/single_session/EMinsight/dep

S2.2. EMinsight multi session expected outputs

The following are representative of the files that are produced by EMinsight to store metadata on a multiple SPA cryoEM experiments

S2.2.1. Multisession collated data files

https://github.com/kylelmorris/EMinsight/tree/publication/expected_outputs/global/csv
